# Mechanical and Rheological Evaluation of Polyester-Based Composites Containing Biochar

**DOI:** 10.3390/polym16091231

**Published:** 2024-04-28

**Authors:** Sebastian Jurczyk, Jacek Andrzejewski, Adam Piasecki, Marta Musioł, Joanna Rydz, Marek Kowalczuk

**Affiliations:** 1 Łukasiewicz Research Network—Institute for Engineering of Polymer Materials and Dyes, 55. M. Skłodowska-Curie St., 87-100 Toruń, Poland; 2 Institute of Materials Technology, Polymer Processing Division, Faculty of Mechanical Engineering, Poznań University of Technology, 3. Piotrowo St., 61-138 Poznan, Poland; jacek.andrzejewski@put.poznan.pl; 3 Institute of Materials Engineering, Faculty of Materials Engineering and Technical Physics, Poznan University of Technology, 3. Piotrowo St., 61-138 Poznan, Poland; adam.piasecki@put.poznan.pl; 4 Centre of Polymer and Carbon Materials, Polish Academy of Sciences, 34. M. Curie-Skłodowska St., 41-819 Zabrze, Poland; mmusiol@cmpw-pan.pl (M.M.); jrydz@cmpw-pan.pl (J.R.); mkowalczuk@cmpw-pan.pl (M.K.)

**Keywords:** biodegradable polymers, biochar, composites, mechanical test

## Abstract

The use of biodegradable polymers as matrices in composites gives a wide range of applications, especially in niche areas. The assessment of the effect of the filler content on the change of mechanical properties makes it possible to optimize the composition for specific needs. Biochar was used as a filler in the studied composites with two different biodegradable blends as a matrix. Poly(1,4-butylene adipate-*co*-1,4-butylene terephthalate)/polylactide/biochar (PBAT/PLA/BC) and polylactide/poly[(*R*)-3-hydroxybutyrate-*co*-4-hydroxybutyrate]/biochar (PLA/P(3HB-*co*-4HB)/BC) composites with 0, 10, 15, 20 and 30 wt% of biochar underwent mechanical tests. The test results revealed a change in the properties of the composites related to the filler content. The results of the tensile test showed that increasing the biochar content increased the tensile modulus values by up to 100% for composites with 30 wt% of biochar, compared to unfilled matrices, and decreased the elongation associated with the breaking of PBAT/PLA and PLA/P(3HB-*co*-4HB) matrix composites. The elongation values at break of PBAT/PLA and PLA/(3HB-*co*-4HB) composites with 30 wt% biochar were reduced by 50% and 65%, respectively, compared to the unfilled matrices. PLA/P(3HB-*co*-4HB) matrix composites, in contrast to PBAT/PLA/BC, showed a decrease in tensile strength with the increases in filler content from 35.6 MPa for unfilled matrix to 27.1 MPa for PLA/P(3HB-*co*-4HB)/BC30 composites. An increase in filler content increased the brittleness of the composites regardless of the matrix used, as determined under the Charpy impact-test. This phenomenon was observed for all tested PLA/P(3HB-*co*-4HB) composites, for which the impact strength decreased from 4.47 kJ/m^2^ for the matrix to 1.61 kJ/m^2^ for the composite containing 30 wt% biochar. PBAT/PLA-based composites with 10 wt% of biochar showed slightly lower impact strength compared to the unfilled matrix, but composites with 30 wt% biochar showed 30% lower impact strength than PBAT/PLA. The complex viscosity value increased with increased filler content. For all composites tested on both polyester matrices, the viscosity decreased with increasing angular frequency.

## 1. Introduction

The mechanical properties of biodegradable composites are important for the performance abilities of products made with them. By introducing fillers into biodegradable matrices, reinforced materials are obtained. If the filler is, additionally, of natural origin, the obtained materials maintain their biodegradability [1]. The type, size and amount of fillers used have colossal impacts on their mechanical properties, leading researchers to attempt to achieve a certain optimal composition, one depending not only on the properties of the final products, but also on the economics of obtaining the products [2,3,4]. The filler used in biodegradable composites, in addition to reducing the price of the final product, can not only improve its mechanical properties, but also fulfil additional functions. The use of biochar as a filler in composites where the matrix was a blend of poly(1,4-butylene adipate-*co*-1,4-butylene terephthalate)/polylactide (PBAT/PLA) has caused a change in the surface resistivity of the obtained materials compared to the pure matrix [5],which definitely extends the application possibilities of this type of composite. Biochar can be viewed as a sustainable approach in biobased polyamide 11 (PA11). Excellent interfacial compatibility between biochar in PA11 matrix, increased tensile strength and modulus of elasticity at tensile test (Young’s modulus), and delayed thermal decomposition processes render a new area of application for this type of material. Composite with 50 wt% of biochar was made appropriate for filament production through additive manufacturing [6]. Additional possibilities are offered by the modification of biochar and the use of other additives, such as silver nanoparticles (AgNP). A steam activation system was used to modify biochar, which was then acid-treated. Composites prepared by the mixing of that biochar with PLA and AgNP were then characterized. They showed properties that made it possible to use them in biosensor development [7]. A spray drying method was utilized to obtain nano-biochar for the preparation of composites with PLA. Additionally, in order to improve the mechanical properties of the material, thymol was used as a PLA plasticizer. With the increase in carbon content in the composites, an increase in electrical conductivity was observed, which means that this type of material can be used as an antistatic packaging. The use of a plasticizer in these composites gave additional application possibilities due to the improvement of the material’s mechanical properties. A lowering of the tensile strength and tensile modulus and an improvement in the elongation were observed for the composites [8]. Composites with biochar can also be obtained by different processing methods. For that purpose, various types of PLA were used to prepare composites with biochar by solvent casting and melt mixing. PLA with a lower average molar mass, due to its better solubility in the solvent used, was selected for the preparation of composites by the solvent-casting method. Specimens after the melt mixing were obtained with a laboratory press. The use of two methods of obtaining composites showed better dispersion of biochar particles in the melt-mixing method. However, there is a significant reduction in the molar mass of the matrix, which is avoided in the solvent-casting method. Rheological behavior indicated a progressive lowering in complex viscosity values with an increasing amount of filler, resulting from the reduction of the molar mass of PLA in the melt-mixing method. The disappearance of the Newtonian behavior observed for the pure matrix was also observed, along with an increase in the amount of biochar in the composite. An increase in the amount of biochar caused the mobility of the PLA chain to slow down due to the interactions between the polymer matrix and the fillers. The case is different for the solvent-casting method, for which the behavior indicates a low level of polymer-filler interactions [9].

Modifications of biochar, various processing techniques, and the use of special additives increase the application possibilities of the composites obtained from the material, but at the same time increase the price of the final products. This is of particular importance when biodegradable polymers are used as the matrix.

PLA, PBAT, and polyhydroxyalkanoates (PHAs) are representative of biodegradable polymers produced on an industrial scale [5,10]. As a brittle material, PLA often requires blending with other polymers in order to increase the range of possibilities for its application. This feature is due to its structure, one with a short repeating unit and a side methyl group. PHAs, due to the possibility of introducing changes in the chain of the repeating unit and modifying comonomer compositions, may have properties along a wide spectrum, from stiff and brittle to ductile/soft and elastic. Commonly used biodegradable polymers of fossil origin include the aliphatic-aromatic copolyester PBAT [11]. Due to its mechanical properties, this polymer is considered the best biodegradable low-density polyethylene (LDPE) equivalent [12]. The blending of biodegradable polymers with each other and use of some additives can wider the application area [13,14].

The use of composites in niche applications requires a series of tests, including mechanical research. Biodegradable composites used for the production of items for special applications must meet certain specific criteria. Earlier studies showed an interesting reduction of the surface resistivity for composites with 30 wt% of biochar in cases where the matrix was a PBAT/PLA blend [5,15].

This publication focuses on the mechanical tests of the previously obtained composites with different compositions, and additionally, for comparative purposes, composites with the same biochar content, manufactured with a polylactide/poly[(*R*)-3-hydroxybutyrate-*co*-4-hydroxybutyrate] (PLA/P(3HB-*co*-4HB)) matrix. The main objective of the presented work is to develop composites based on a biodegradable polymer matrix with carbonized biomass material used as filler to evaluate the material’s mechanical and rheological properties. The analyses were complemented by microstructural observations using the SEM method. Biochar used as filler is also an attractive material, from the point of view of carbon fixation technology, as a bio-based material. This evaluation of the mechanical properties will help to find potential application areas for the tested composites.

## 2. Materials and Methods

### 2.1. Materials

Ecovio^®^ F Mulch C2311 (PBAT/PLA), BASF Company (Florham Park, NJ, USA), a commercial blend of PBAT (47 mol% of aromatic segments) with 25 mol% of PLA (determined by nuclear magnetic resonance spectroscopy), as described in previous work [5], and SOOGREEN 2001a (PLA/P(3HB-*co*-4HB)) with 12% of 4-hydroxybutyrate (4-HB) units in P(3HB-*co*-4HB), 78% PLA units in blend, and 1 mol% plasticizer acetyl tributyl citrate (ATBC) (calculated by proton nuclear magnetic resonance spectroscopy), from Tianjin GreenBio Materials (Tianjin GuoYun Biological Material Co., Ltd., Tianjin, China), were utilized as a matrix for composites with different amounts (10, 15, 20 and 30 wt%) of biochar. PBAT/PLA blends and (PLA/P(3HB-*co*-4HB)) blends have been reported in several publications [16,17,18,19].

Biochar from Fluid S.A. (Sędziszów, Poland) was used as filler in the preparation of the composites. For the preparation of the biochar, the wood chips of conifer trees were used. The material was subjected to a pyrolysis process, during which the temperature reached 650 °C. Before being used as the polymer filler, the raw BC was subjected to a grinding procedure which used the ball milling method. According to the specifications provided by the manufacturer, the elemental carbon content of the discussed biochar variety is approximately 80%. During the thermogravimetric analysis (TGA) of the biochar in nitrogen, two mass loss steps were observed. The first was related to water evaporation and the second to biochar decomposition (see Figure S1 in the Supplementary Materials). The decomposition process started at around 400 °C, when 5% of the sample mass was decomposed, and the residual content was around 83% at 800 °C. Due to the presence of water in the filler, the biochar was dried before preparation of the composites.

### 2.2. Preparation of Composites

**Figure 1:**
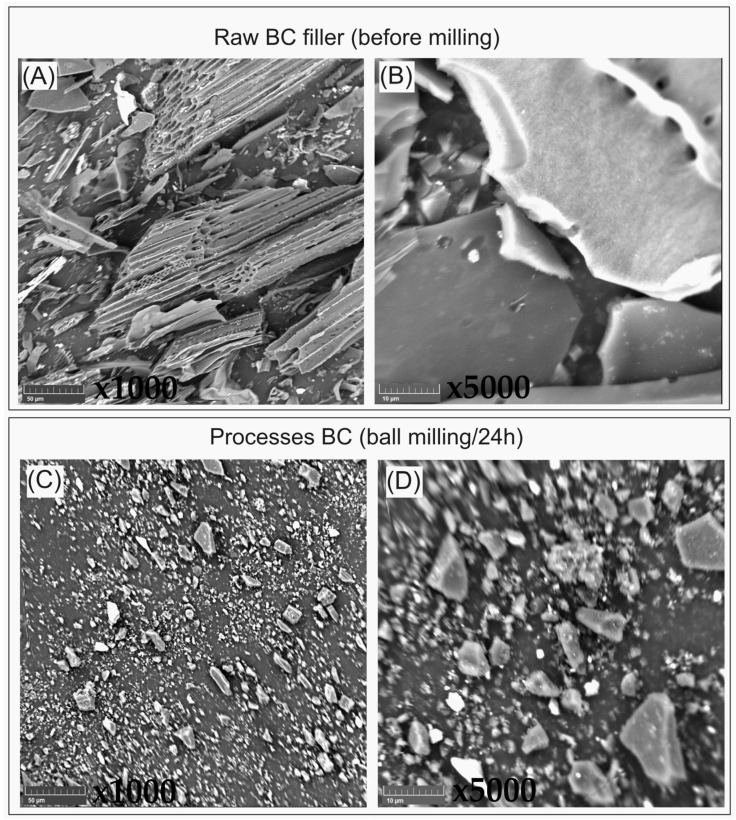
SEM micrographs presenting the (**A**,**B**) raw biochar (BC) filler and (**C**,**D**) ball-milled filler with reduced particle sizes.

Due to the large size of the raw biochar particles, the filler was subjected to a grinding procedure with the use of the ball milling method. For this purpose, stainless steel containers were used, and 10 mm steel balls were used as milling media. The process was conducted for 24 h at 60 rpm. The process was carried out using a roller mill machine (model Metalchem, Lodz, Poland). The results of the conducted ball milling procedure are presented in Figure 1, where the SEM micrograph of the raw and ball-milled BC particles are revealed. The average size of the processed BC filler particles was reduced to around 1.5 µm, while the size of the untreated particles ranged from 100 µm to 2 mm. The full procedure of ball milling has already been described in our previous work [5], while the properties of the ball-milled biochar particles have also already been investigated in many studies [20,21,22].

Before melt processing, all compounds were dried using a cabinet oven. For all materials the procedure was conducted at 60 °C for 24 h; after that, the dry blends were prepared. Polymer pellets were mixed with biochar at four different concentrations of filler: 10, 15, 20 and 30 wt%. A kitchen rotary mixer was used for the preparation of the initial mixtures. The main blending procedure was conducted using twin-screw extruder, model Zamak EH16D (Zamak Mercator, Skawina, Poland). The extrusion process was carried out with a maximum temperature of 160 °C, and the screw speed was set to 100 rpm. The extruded material was cooled and pelletized.

The prepared pellets were used for the manufacturing of standardized specimens using the injection molding technique. The molding process was performed using an Engel e-mac 50 machine (Engel Austria GmbH, Schwertberg, Austria), for which the injection temperature was set to 175 °C, the injection/holding pressure was 1050/550 bar, the holding/cooling time was 15/45 s and the mold temperature was 40 °C. Afterwards, the molding specimens were conditioned in a sealed bag (20 °C, 48 h). The detailed list of parameters of the extrusion, injection molding process and full procedure of blending and injection molding has already been described in our previous work [5].

### 2.3. Methods

#### 2.3.1. Fourier Transform Infrared Spectroscopy (FTIR)

Changes in the structure and the surface of the material were analyzed by Fourier transform infrared (FTIR) using an FT/IR-6000 Jasco instrument (Jasco Corporation, Tokyo, Japan). The spectra were analyzed at a high resolution of 4 cm^−1^, covering a wide range from 4000 to 200 cm^−1^, and the data were averaged over 64 scans.

#### 2.3.2. Surface Morphology

Scanning electron microscopy (SEM) observations were performed using a Tescan MIRA3 apparatus (Tescan, Brno, Czech Republic). All images were obtained from the fractured surfaces of the impact-strength specimens. Before scanning, the investigated surface was covered with a thin layer of carbon coating (≈20 nm). For most specimens, the SEM microscope was working with an accelerating voltage of 12 kV.

#### 2.3.3. Mechanical Test

The mechanical properties of the prepared blends and composites were examined using a static tensile test and the Charpy impact-strength test. The tensile properties were measured using a Zwick/Roell Z010 universal testing machine (Zwick/Roell GmbH, Ulm, Germany). The measurements were performed according to the ISO 527-1 standard [23], using 1A-type specimens (4 mm thickness). The initial force for samples with the PBAT/PLA matrix was set to 2 N, while for stiffer samples with the PLA/P(3HB-*co*-4HB) matrix it was increased to 5N. The crosshead speed was set to 50 mm/min. A minimum of 5 specimens were used for the single test series.

Impact-resistance tests were performed using a Zwick/Roell HIT15 testing machine (Zwick/Roell GmbH, Ulm, Germany). The apparatus was equipped with a 5 J pendulum. The Charpy notched impact-strength test was conducted in accordance with the ISO 179-1 standard [24]. Rectangular specimens (80 mm × 10 mm × 4 mm) were notched in the middle section of the bar, with an A-notch depth of 2 mm. The direction of blow was edgewise. A minimum of 10 specimens were used for the single test series.

#### 2.3.4. Dynamic Rheology

Rheological measurements were conducted using an Anton Paar MCR301 rheometer (Anton Paar GmbH, Graz, Austria). The apparatus was equipped with a 25 mm plate–plate system with the gap distance set at 1 mm. All tests were performed using the small-amplitude oscillatory shear method. The deformation strain was 1%, and the deformation frequency ranged from 0.1 to 500 rad/s. All measurements were conducted at 190 °C. Before the main frequency sweep analysis, all materials were tested using amplitude sweep measurements in order to determine the linear viscoelastic range. The results of the frequency sweep measurements are presented in the form of storage modulus, loss modulus and complex viscosity plots.

## 3. Results

### 3.1. Structural Analysis

**Figure 2:**
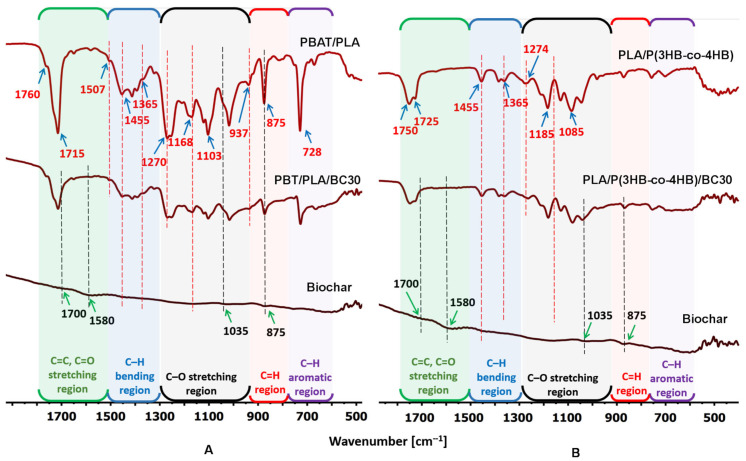
Selected representative FTIR spectra of the (**A**) PBAT/PLA and (**B**) P(3HB-*co*-4HB), along with biochar and the associated composite with 30 wt% of biochar.

The structural analysis of composites based on PBAT/PLA or PLA/P(3HB-*co*-4HB) and the interactions between the polymer matrix and the filler were carried out using the FTIR method (Figure 2). No significant changes were observed in the structures of the investigated materials.

The FTIR spectra of PBAT/PLA and PLA/P(3HB-*co*-4HB) show characteristic stretching frequencies for the following: (i) C=O at 1760 cm^−1^ in PBAT/PLA and 1750 cm^−1^ in PLA/P(3HB-*co*-4HB) for PLA, as well as 1715 cm^−1^ for PBAT and 1725 cm^−1^ for P(3HB-*co*-4HB); (ii) C–O of the –CH–O– moiety at 1184 cm^−1^ for PLA/P(3HB-*co*-4HB) and 1168 cm^−1^ for PBAT; and (iii) C–O of the O=C–O moiety at 1103 cm^−1^ for PBAT and 1120 cm^−1^ for PLA in PBAT/PLA, as well as 1085 cm^−1^ for PLA and 1045 cm^−1^ for P(3HB-*co*-4HB) in PLA/P(3HB-*co*-4HB). The bending frequencies for asymmetric and symmetric deformation vibration of the –CH_3_ moiety are seen at 1455 (also –CH_2_– bending for PBAT) and 1365 cm^−1^ for PLA and P(3HB-*co*-4HB), respectively. The band at 728 and 1507 cm^−1^ represents the external bending vibration absorption signal of the C–H moiety and the stretching vibration of C=C in the PBAT aromatic ring. The signal at 875 cm^−1^ is the result of para-substitution of the benzene ring [25,26].

The FTIR spectrum of biochar shows signals characteristic of a stretching frequency at 1700 cm^−1^ for the carbonyl groups of carboxyl moieties, C=O stretching and aromatic C=C frequency at 1580 cm^−1^ and signal out-of-plane deformation produced by the aromatic C−H moiety at 875 cm^−1^ [27]. The intensity of the polymer matrix bands decreases with the increases in biochar content in the composite.

### 3.2. Surface Characteristics

**Figure 3:**
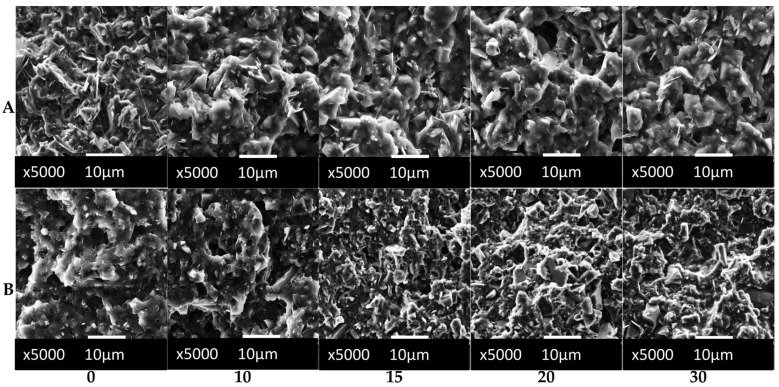
SEM micrographs of (**A**) PBAT/PLA-based and (**B**) PLA/P(3HB-*co*-4HB)-based composites with 0, 10, 15, 20 and 30 wt% of biochar.

Microscopic analysis of the composites prepared with biochar was carried out using SEM. The fractures of the specimens after the impact tests were analyzed. Figure 3 shows a summary of the obtained SEM micrographs.

Detailed structural observations of the PBAT/PLA and the PLA/P(3HB-*co*-4HB) composites revealed the presence of holes and pull-outs of filler particles on the surfaces of the fractures. The filler particles were observed to have a porous structure similar to that of a cellular structure, characterized by open pores that remained void.

The observed porosity of composites, especially for the PLA/P(3HB-*co*-4HB) matrix, is one of the main reasons for the brittleness of these materials, which is related to the initiation of crack propagation in the filler particles. In the case of biochar, one way to reduce the number of pores might be the biochar milling process, which is reported by Gezahegn et al. [28]. The matrix–biochar interaction is mainly provided by physical interactions and depends on the conditions of biochar production.

The conditions under which biochar is produced, especially the temperature during production, have an impact on the development of the surface area of this filler. The most important factor is the size of the filler particle. According to reports in the literature [29,30,31], the smaller the particle size of the biochar filler, the better the composite reinforcing effect observed. This is due to the interfacial interactions due to the more advanced surface development of the filler particles.

Both composites, containing 10 and 30 wt% biochar, showed the presence of filler particles in the matrix on the analyzed fracture surfaces. In particular, the composite containing 30 wt% biochar showed filler particles as dominant on the breakthrough/fracture surface. The results of the mechanical tests and the analysis of the breakthrough/fracture surface indicate that the filler surface is not sufficiently wetted by the matrix, indicating a lack of chemical interactions at the phase (filler-matrix) interface [32].

The hydrophilic nature of most polymers limits the adhesion of natural fillers. This phenomenon is related to the presence of functional groups on the surface of the filler particles, mainly carboxyl and hydroxyl groups. One way to improve the adhesion of the polymer matrix to biochar is to modify the filler surface by using compatibilizers i.e., coupling agents that increase the polarity of the polymer matrix. For example, maleic anhydride can be used for this purpose [33]. The surface appearance of voids/fractures in PBAT/PLA composites with biochar indicates poor wetting of the filler surface by the matrix.

### 3.3. Mechanical Tests

**Figure 4:**
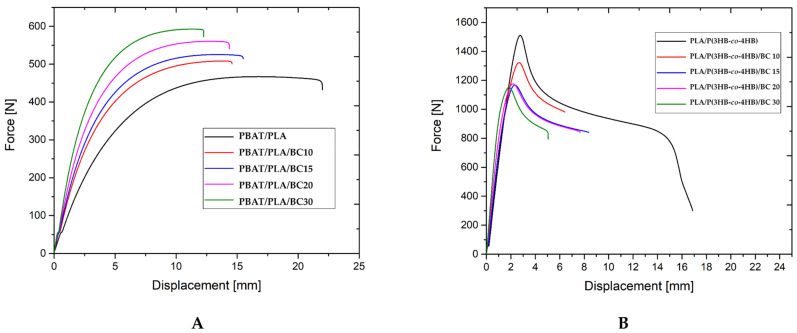
Plots presenting the tensile test curves (force/displacement): (**A**) for PBAT/PLA-based composites and (**B**) for PLA/P(3HB-*co*-4HB)-based composites.

The mechanical tests at static tension were carried out to determine yield stress, tensile strength, elongation-at-yield and at break, and tensile modulus. The tensile test curves (force vs. displacement) for PBAT/PLA/biochar (BC) and PLA/P(3HB-*co*-4HB)/BC composites are presented in Figure 4.

**Figure 5:**
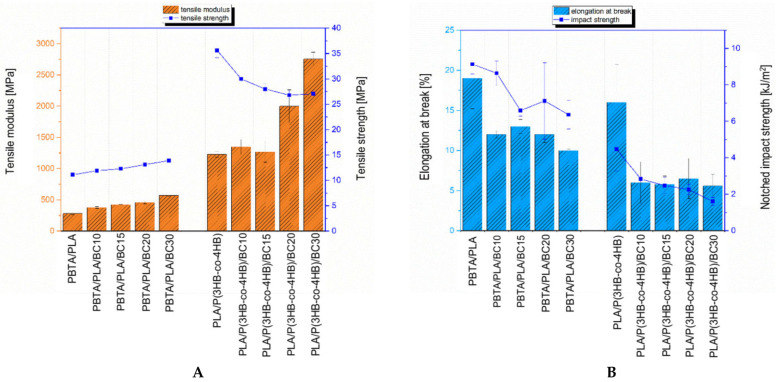
The results of static tensile tests and Charpy impact-resistance measurements. Results are presented in the form of (**A**) tensile modulus/strength plots and (**B**) elongation-at-break/impact-strength results.

The fracture of the specimens occurred when the yield stress was exceeded (Figure 4). The elongation-at-break decreased with increasing filler content for composites for both polymer matrices, as presented in Figure 5.

Composites containing 10, 15 and 20 wt% of biochar had similar values for elongation-at-break, ranging from 12 to 13%. The composite containing 30 wt% biochar filler achieved the lowest elongation at break, of 10%. However, it should be noted that the standard deviation as presented in Table S2 in the Supplementary Material was very low (0.15%), which may be related to the good distribution of the filler in the matrix [34]. The 30 wt% PBAT/PLA/biochar composite showed half the elongation value of the pure matrix, which reached an average value of 19%. A similar trend in the results for elongation-at-break versus filler content was observed for elongation-at-yield. Elongation-at-yield values are given in Table S2 in the Supplementary Material. The highest elongation-at-yield was achieved by the PBAT/PLA matrix and was 14%. The composite containing 10 wt% biochar showed an elongation-at-yield of 11%, and the composites containing 15 and 20 wt% filler had similar elongations, of about 9.5%. The lowest elongation-at-yield value, 7.7%, was obtained from a composite containing 30 wt% of biochar. The use of a biochar filler increased the stiffness of the composites compared to the pure matrix, with the tensile modulus increasing with increasing filler content. The 30 wt% biochar content doubled the tensile modulus value relative to the value obtained for the matrix. Composites with a lower biochar content showed tensile modulus values intermediate between the pure matrix and the composite with the highest filler content. The tensile modulus value increased with increasing filler content. The standard deviation values of the analyzed results were low with respect to the mean value, indicating good preparation of the specimens, which showed an almost proportional dependence between the increase of the tensile modulus value and increasing filler content in the composite. The use of a biochar filler increased the stiffness of the composites relative to the pure matrix, with the tensile modulus increasing with increasing filler content. The 30 wt% biochar content doubled the tensile modulus value relative to that obtained for the pure polymer matrix. Composites with a lower biochar content showed modulus values intermediate between the pure matrix and the composite with the highest filler content. The tensile modulus value increased with increasing filler content. The standard deviation values of the analyzed results were low with respect to the mean value, indicating good specimen preparation. An almost proportional dependence between the increase in the tensile modulus value and increasing filler content in the composite was observed.

The tensile strength value for the composites was higher than that for the PBAT/PLA pure matrix and increased with increasing filler content (Figure 5A). It should be noted that the increase in the strength of the composite containing 30 wt% biochar was 25% relative to that of the pure matrix. The observed dependence can be explained by considering several aspects. The first is a higher content of PBAT, compared to the levels associated with the PLA, within the polymer matrix, which means that the material underwent greater deformation. Another is the size of the filled particle, which is on average 1.5 µm, as achieved by an effective grinding process. The micrographs of biochar shown in Figure 1, after ball milling, show a filler with a defined particle size. Due to the use of a well ground filler without large particles and appropriate homogenizing in the polymer matrix (Figure 2A), this filler did not weaken the composite. Based on the test results described for the PBTA/PLA (Ecovio^®^) composites presented in our previous work [5], the effect of increasing the tensile strength is also associated with increasing the crystallinity of the polymer matrix in the composites, which was confirmed by differential scanning calorimetry (DSC) and X-ray diffraction (XRD) studies, in which the degree of crystallinity was calculated to be 16% for the unfilled matrix and 19% for the 30 wt% biochar composite. Furthermore, the DMA studies showed that the flexible segment of the PBAT/PLA matrix dominates. Composites containing 10 and 15 wt% biochar showed tensile strengths at a similar level. These two composites have favorable mechanical properties among the specimens tested. They are stronger in tension tests, compared to the pure matrix, and more rigid (the Young’s modulus of the 15 wt% composite is about 50% higher than that of the pure matrix), but not so rigid and brittle as the 30 wt% biochar composite, while showing the potential to achieve relatively high deformation. Georgiopoulos et al. [35] reported mechanical properties of PBAT/PLA composites with 2, 3 and 5 wt% of silica nanofiller and with 20 and 30 wt% of wood-flour obtained from softwood. The tensile strength for composites with 3 and 5 wt% of silica nanofillers increased but 2 wt% of silica decreased tensile strength. Wood-flour filler decreased tensile strength for the tested composite. Both fillers reduced the tensile modulus value regardless of the filler content in the composites.The results reported for wood-flour composites are similar to the results in this paper, which suggests that biochar can play the role of an efficient, low-cost filler for PBAT/PLA composites, in place of the wood-flour.

**Figure 6:**
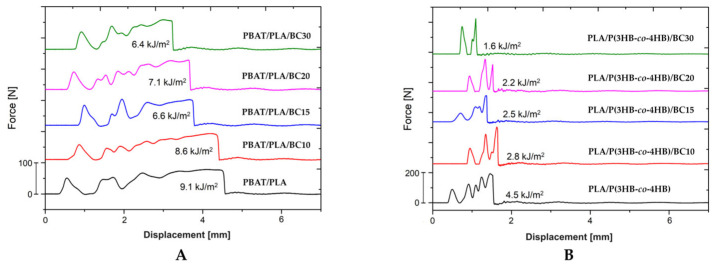
Impact load measurements recorded during the Charpy impact-strength tests. Force/displacement curves for (**A**) PBAT/PLA-based composites and (**B**) PLA/P(3HB-*co*-4HB)-based composites.

The impact strength of the PBAT/PLA composites decreased as the amount of biochar increased relative to the pure matrix. Compared to the unfilled PBAT/PLA matrix, the 10 wt% biochar composite showed the least reduction in Charpy impact-strength. (Figure 5B and Figure 6).

When the filler content was increased, the impact strength was significantly decreased, by 30% for the 15 wt% biochar composite relative to the pure matrix. It should be noted that when comparing the impact strength of composites containing 15 and 30 wt% biochar, the mean Charpy impact-strength values for these notched specimens are only slightly different (Figure 6). The 20 and 30 wt% biochar composites have the greatest variation in results, as determined by the standard deviation values, among the PBAT/PLA-based composites. The 30 wt% biochar composite was the most brittle. The standard deviation values of the Charpy impact-test are given in Table S3 in the Supplementary Material. The Charpy impact-strength results for these composite/filler concentrations show a good relation with the tensile test results, in which it was found that that the elongation was the lowest and the tensile modulus was the highest among the PBAT/PLA-based composites tested (Figure 5).

The PLA/P(3HB-*co*-4HB)-based composites exhibited good strength-at-yield in the tensile test (Figure 4). Fracture occurred when the strain reached several times the strain recorded at the yield stress. During the initial phase of the tensile test, the stress increased in proportion to the induced strain until the yield point was reached. Once the yield point was exceeded, the stress decreased, and the polymer matrix continued to deform until the specimen fractured. The instance of this behavior characterized by the highest deformation was observed for the pure matrix. The biochar composites showed lower elongation-at-break values than the pure PLA/P(3HB-*co*-4HB) matrix, and also lower elongation-at-break values than those recorded for PBAT/PLA-based composites containing the same biochar concentration (Figure 5). The mentioned change in elongation values had previously been observed for the composite with the lowest filler content (10 wt%). As presented in Figure 4, the elongation-at-yield point of the 10% biochar composite was similar to that of the pure PLA/P(3HB-*co*-4HB) matrix. With increasing filler content, the composites show lower elongation. The elongation-at-yield obtained for the PLA/P(3HB-*co*-4HB)-based composites is very repeatable, as shown by the low standard deviation value (see Table S2 in the Supplementary Material). The tensile modulus values of the composites containing 10 and 15 wt% biochar are similar to that determined for the pure PLA/P(3HB-*co*-4HB) matrix. It should be noted that the standard deviation of the determined value is higher for the composites than for the pure matrix, which may indicate the effect of the filler in increasing the stiffness of the composites. Significant increases in stiffness were observed for the composites with a higher filler contents, of 20 and 30 wt%. The 30 wt% biochar composite had more than twice the tensile modulus of the pure matrix, 2756 MPa and 1230 MPa, respectively, for the 30 wt% composite and pure matrix. The 20 wt% biochar composite had a modulus of stiffness of 2000 MPa, which was significantly higher than those for composites with lower filler contents. The tensile modulus value for this composite was intermediate between the result for the 15 wt% biochar composite and for the 30 wt% biochar composite. The standard deviation for the tensile modulus of the 20 wt% composite (see Table S1 in the Supplementary Material) was the largest of the tested specimens, and was 13% of the mean value. The large deviation may indicate the significant effect of the filler content used on the linear deformation properties of the composite. Loureiro et al. [36] reported tensile tests results for PLA/P(3HB-*co*-4HB) composites with 10, 20 and 30 wt% of cellulosic fiber, showing that tensile strength and tensile modulus increased with increases in filler content. The highest values were reported for 30 wt% composites. The authors reported that the elongation-at-yield values decreased with increasing filler content. When comparing the tensile results presented in this paper for biochar filler with cellulosic filler, similar tendencies are seen for tensile modulus and the elongation-at-yield value. The differences in the tensile strength results can be attributed to the form of biochar filler, which was prepared by the grinding process using a ball milling method. The obtained biochar particles are being compared to a filler in the form of a flour; biochar does not strengthen composites within the PLA/P(3HB-*co*-4HB) matrix in this case.

The tensile strength of the PLA/P(3HB-*co*-4HB) biochar composites decreased with increases in the filler content of the composite. The observed behavior of PLA/P(3HB-*co*-4HB) matrix composites is different from that recorded for composites with PBAT/PLA matrix, for which the tensile strength value increased with increases in the biochar content in the composite. The lack of occurrence of the desired and expected reinforcing effect of the biochar filler may have been caused by the lack of sufficient filler adhesion to the matrix [25]. The deterioration in tensile strength for the composites, compared to the strength of the pure matrix, can be seen from the results of elongation-at-break results, which were significantly reduced, compared to the pure matrix [37]. The composites with 15 wt% and higher biochar content showed similar tensile strength values. The reported difference in mean value between these composites was 1 MPa, which was 3.7% of the mean value. PLA/P(3HB-*co*-4HB)-based composites with biochar showed no strengthening effect.

The impact strength of the PLA/P(3HB-*co*-4HB) composites decreased relative to the pure matrix, which was similar to results seen for the PBAT/PLA matrix biochar composites (Figure 5). The impact strength decreased with increasing filler content in the composites. The 20 wt% biochar composites showed half the impact strength of that reported for the pure matrix. The composite with the highest filler content had a lower impact strength, by about 60%, compared to the matrix. Such large changes in the reduction of the impact strength were not observed for the PBAT/PLA composites. It should be noted that the results for impact strength for the PLA/P(3HB-*co*-4HB) correlate with the results for mechanical properties determined in the tensile mode test (tensile modulus, tensile strength, and elongation-at-break), i.e., as the biochar content increased, stiffness increased and tensile strength and elongation-at-break decreased. Collectively, these results indicate that biochar in PLA/P(3HB-*co*-4HB) composites may require additional treatments to enhance matrix–filler interactions, e.g., compatibilization with the matrix using coupling agents. Otherwise, it is expected that PHA- and PLA-based composites will exhibit poorer mechanical properties than the pure matrix.

The surface appearance of the fractures (Figure 3) is similar to that described in the literature for biochar composites with a thermoplastic matrix of petrochemical origin, e.g., polypropylene (PP) or polyoxymethylene (POM), which did not show chemical interactions at the interface. The level of filler-matrix interactions is higher in the PBAT/PLA matrix than in the PLA/P(3HB-*co*-4HB) matrix, which was confirmed in mechanical tests where the tensile strength increased with increasing filler content, along with a reduction in impact strength of approximately 30%, which was not as significant as in the case of the PLA/P(3HB-*co*-4HB) matrix. The reduction in impact strength of the PLA/P(3HB-co-4HB)-based composite with 30 wt% of biochar was three times that of the pure matrix (see Figure 5).

Based on the results of the impact-strength tests (see Figure 5B), it can be concluded that the particle size of the biochar used in the composites was large enough to have a negative effect, increasing the brittleness of the material and decreasing the impact strength due to crack propagation through the entire cross-section of the specimen. The reduction in impact strength was observed for the composites in both matrices used, and it was greater where the proportion of filler content in the specimen was higher. It should be noted that no chemical modification of the filler surface was used, a factor which also did not increase the impact strength of the composites.

### 3.4. Dynamic Rheology

**Figure 7:**
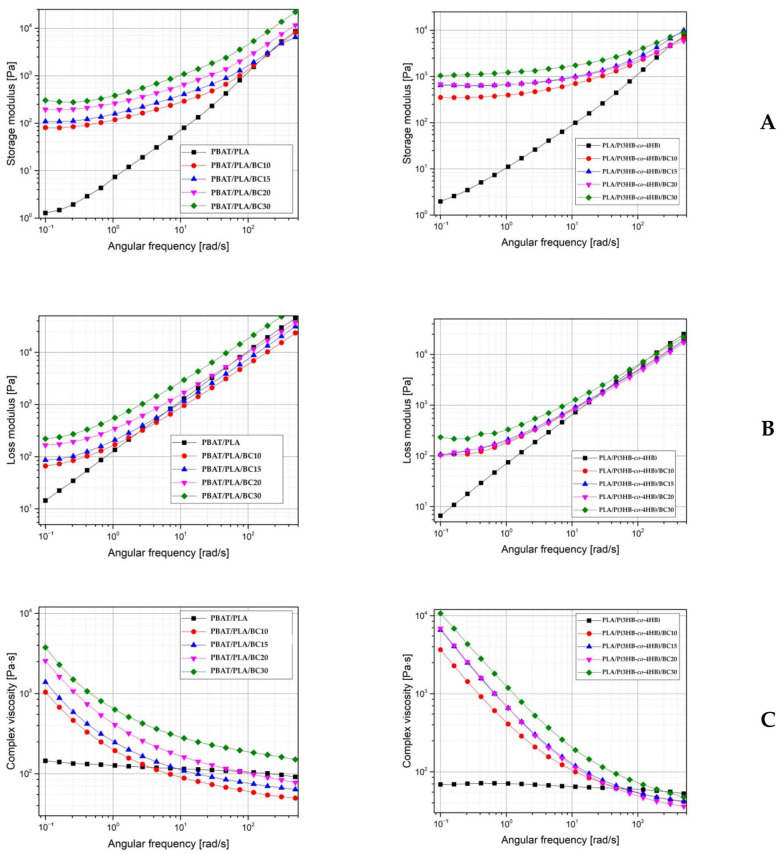
The results of small-amplitude oscillatory shear tests. Storage modulus (**A**), loss modulus (**B**) and complex viscosity (**C**) plots for PBAT/PLA- and PLA/P(3HB-*co*-4HB)-based materials.

Rheological characteristics of the tested composite materials were determined by small-amplitude oscillatory shear tests. The linear viscoelastic region was determined, and the first phase of the measurements was carried out in frequency sweep mode. The results of the complex viscosity (*η*), loss modulus (*G*″) and storage modulus (*G*′) tests are presented in Figure 7. The influence of the filler content is noted for both the PBAT/PLA and the PLA/P(3HB-*co*-4HB) matrices. The presence of physical interactions between the filler particles always has a meaningful influence/effect on the rheological characteristics.

The presence of the filler increased the viscosity of the composites relative to the pure matrix, and this change is particularly evident at a low angular frequency. Similar behavior is exhibited by both of the used matrices, as shown in the chart showing the change in composite viscosity (Figure 7C). The change in viscosity is reported for all the composites tested; it is particularly well seen at low angular frequency values and involves changes in the storage modulus and the loss modulus. The post-processing specimens showed no signs of degradation at low angular frequency values. The results of the rheological tests show a decrease in viscosity with increasing angular frequency. Above 100 rad/s, the viscosity of the PLA/P(3HB-*co*-4HB) composites was at a level similar to that of the pure matrix.

For the measurement range of 4 to 400 rad/s, the values of complex viscosity for PBAT/PLA composites with 10 and 15 wt% biochar are lower than those for the pure matrix. The same phenomenon was also observed for PLA/P(3HB-*co*-4HB) composites above 100 rad/s. In contrast to the fact that no visual signs of degradation were observed during injection processing, the results of dynamic rheology tests expressed by the complex viscosity value may indicate a decrease in the molecular weight of the polymer matrices. The reduction in complex viscosity value observed for PBTA/PLA 10 and 15 wt% biochar composites in the measurement range above 4 rad/s may indicate the occurrence of the degradation phenomenon in these samples. The observed change in viscosity for these two PBTA/PLA composites above an angular frequency of 4 rad/s and the PLA/P(3HB-*co*-4HB) composites at the range above 100 rad/s, which was revealed as lower viscosity values compared to those determined for pure matrices, may be related to the initiation of the degradation of these materials, as revealed after the exceeding of certain angular frequencies, which were different for each polymer matrix. Analyzing the storage modulus values, which are higher for composites than for pure matrices, it was observed that the degradation limit decreases with increasing angular frequency. This behavior is also confirmed by the change in loss modulus values, which are higher for composites than for matrices. PLA/P(3HB-*co*-4HB) composites are more stable, in terms of the viscosity tests. The viscosity of pure matrices is not very high. The addition of biochar changes the viscosity, increasing it, compared to pure polymer matrix. The filler slows down the mobility of the polyester chains due to matrix-filler interactions.

The addition of biochar filler to the PBAT/PLA and PLA/P(3HB-*co*-4HB) matrices caused rheological changes which are very similar to those observed for spherical fillers such as talc [20].

The moisture content in a biochar is important for its rheological properties. Typically, the moisture content in biochar is around 6–10%. After drying, the moisture content is lower (below 1%), but in this case the water is responsible for hydrolytic degradation, which is observed in polyester matrices.

## 4. Conclusions

In the present study, composites of PBAT/PLA and PLA/P(3HB-*co*-4HB) blends with biochar were prepared and evaluated during mechanical tests, specifically, the static tensile test and the Charpy impact-strength test. Small-amplitude oscillatory tests were also performed. The addition of biochar as a filler in the composites with the mentioned blends had a visible influence on the properties determined in the tensile tests, especially on elongation-at-break, which decreased with increases in the biochar concentration of the material. The elongation-at-break was reduced 50% for PBAT/PLA and 65% for PLA/(P3HB-*co*-4HB) composites with 30 wt% of biochar, compared to the pure matrices. In the case of the PLA/P(3HB-*co*-4HB) matrix, a significant change in elongation-at-break, close to that achieved by the 30 wt% composite, is already observed at the lowest filler content of 10 wt%. The stiffness of prepared composites was higher than values seen in pure blends, and samples were more rigid with increasing filler content. The tensile modulus values for composites with 30 wt% of filler were twice those of the filler-free matrices. The tensile strength of PLA/P(3HB-*co*-4HB) biochar composites decreased with increased filler content in the composite, from 35.6 MPa to 26.8 and 27.1 MPa for composites with 20 and 30 wt% of filler, respectively. The PBAT/PLA-based composites demonstrate different strength parameters, compared to the PLA/P(3HB-*co*-4HB) composite, by increasing tensile strength with increasing biochar content, from 11.1 MPa for the matrix to 13.9 MPa for composite with 30 wt% of filler. A similar effect of filler on the reduction of impact strength was observed for both polymer blends used. PBAT/PLA-based composites with 10 wt% biochar showed slightly lower impact strength, compared to unfilled matrix, specifically, 8.64 and 9.13 kJ/m^2^, respectively. Composites with 30 wt% biochar showed lower impact strength than the PBAT/PLA matrix, a reduction of about 30%, to 6.36 kJ/m^2^. Reduction of impact strength was observed for all tested PLA/P(3HB-*co*-4HB) composites. The tested value decreased from 4.47 kJ/m^2^ for the matrix to 1.61 kJ/m^2^ for the composite containing 30 wt% biochar, and it was a more significant reduction of impact strength than seen for the PBAT/PLA matrix. The addition of biochar changes the viscosity by increasing it, compared to pure matrix. The biochar slows down the mobility of the polyester chains due to matrix-filler interactions. These phenomena were observed for value changes of certain determined parameters, such as storage modulus, loss modulus, and complex viscosity. In the cases of both polymer matrices, the composites achieved higher values with increases in the filler content. The most significant change of complex viscosity value was for PLA/P(3HB-*co*-4HB) composites with 30 wt% of filler, from 1000 Pa·s at 10^−1^ rad/s to a value of 60 Pa·s at 110 rad/s, which was equal to that achieved by the unfilled matrix. Although the PLA/P(3HB-*co*-4HB) matrix has a higher tensile strength than PBAT/PLA, both it and its composites exhibit lower elongation-at-break and lower impact strength/more brittleness. The above relationships have implications for the future application of the composites tested in terms of the requirements placed on them. One of the two biochar composite matrices tested could be used, depending on whether the biocarbon composite is required to have a higher tensile strength but lower ductility, or a lower tensile strength but higher ductility and impact resistance. The overall conclusion of the research is that the biocomposites presented in this paper, which use biochar as a filler and polyester blends as a matrix, could be materials for new applications, i.e., in the packaging, automotive and electronics industries, for small-sized products for which it is not necessary to use engineering plastics, and it is important to increase the proportion of polymeric materials from natural sources in relation to commercial scale of production. Biochar has a lower cost and is derived from biomass. In this context, there has been an exponential increase in the number of studies on the formulation of polymer systems containing biochar, which are usually based on thermoplastic matrices. In view of its interesting properties and the possibility of tailoring its structure and functionalization, biochar is an attractive alternative to traditional carbonaceous fillers for improving the mechanical, electrical and physical properties of polymer-based composites. The biochar induces particularly beneficial changes in PBAT/PLA composites, stiffening and strengthening them.

## Data Availability

The raw/processed data required to reproduce these finding are available upon request.

## Supplementary Materials

The following supporting information can be downloaded at: https://www.mdpi.com/article/10.3390/polym16091231/s1, Figure S1. TGA and DTG curves of the biochar; Table S1. Results of determination of tensile modulus and tensile strength; Table S2. Results of determination of elongation at yield and elongation at break; Table S3. Results of determination of Charpy impact strength of notched specimens.
