# Supplementary material for: Mechanical and Rheological Evaluation of Polyester-Based Composites Containing Biochar
Source: Polymers (Basel). 2024 Apr 28;16(9):1231. doi: 10.3390/polym16091231 (PMC11085265; doi:10.3390/polym16091231)
Supplement: Supplementary file 1 [file polymers-16-01231-s001.zip › polymers-2961658-supplementary.pdf]

Supplementary materials

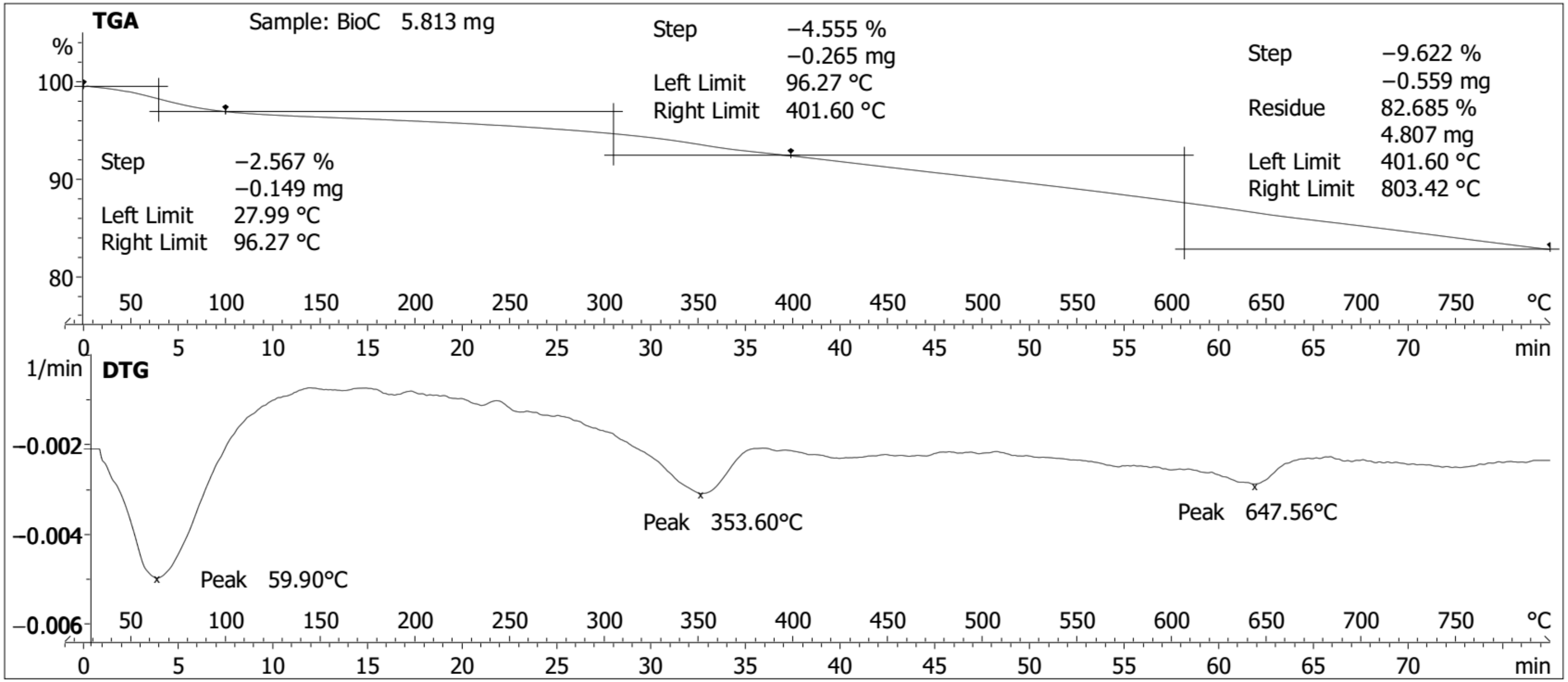

Figure S1. TGA and DTG curves of the biochar.

Table S1. Results of determination of tensile modulus and tensile strength

Specimen	Tensile modulus E_t [MPa]			Tensile strength R_m [MPa]	
	Mean value	Standard deviation		Mean value	Standard deviation
**PBAT/PLA**	**281**	**16.8**		**11.1**	**0.10**
**PBAT/PLA BC10**	**377**	**14.3**		**11.9**	**0.07**
**PBAT/PLA BC15**	**422**	**7.43**		**12.3**	**0.08**
**PBAT/PLA BC20**	**457**	**19.7**		**13.1**	**0.03**
**PBAT/PLA BC30**	**573**	**21.2**		**13.9**	**0.10**
**PLA/P(3HB-*co*-4HB)**	**1230**	**45.8**		**35.6**	**1.41**
**PLA/P(3HB-*co*-4HB) BC10**	**1350**	**118**		**30.0**	**0.26**
**PLA/P(3HB-*co*-4HB) BC15**	**1270**	**169**		**28.0**	**1.10**
**PLA/P(3HB-*co*-4HB) BC20**	**2000**	**264**		**26.8**	**0.80**
**PLA/P(3HB-*co*-4HB) BC30**	**2756**	**110**		**27.1**	**0.50**

Table S2. Results of determination of elongation at yield and elongation at break

Specimen	Elongation at yield ε_y [%]			Elongation at break ε_b [%]	
	**Mean value**	**Standard deviation**		**Mean value**	**Standard deviation**
**PBAT/PLA**	**14.0**	**0.38**		**19.0**	**3.80**
**PBAT/PLA BC10**	**11.0**	**0.23**		**12.0**	**0.42**
**PBAT/PLA BC15**	**9.8**	**1.10**		**13.0**	**0.86**
**PBAT/PLA BC20**	**9.5**	**1.10**		**12.0**	**1.00**
**PBAT/PLA BC30**	**7.7**	**0.51**		**10.0**	**0.15**
**PLA/P(3HB-*co*-4HB)**	**2.3**	**0.08**		**16.0**	**4.70**
**PLA/P(3HB-*co*-4HB) BC10**	**2.2**	**0.10**		**6.0**	**2.60**
**PLA/P(3HB-*co*-4HB) BC15**	**2.0**	**0.04**		**5.8**	**1.00**
**PLA/P(3HB-*co*-4HB) BC20**	**1.8**	**0.09**		**6.5**	**2.50**
**PLA/P(3HB-*co*-4HB) BC30**	**1.5**	**0.09**		**5.6**	**1.40**

Table S3. Results of determination of Charpy impact strength of notched specimens

Specimen	Charpy impact strength a_{cN} *[kJ/m²]*	
	Mean value	Standard deviation
**PBAT/PLA**	**9.13**	**0.53**
**PBAT/PLA BC10**	**8.64**	**0.67**
**PBAT/PLA BC15**	**6.59**	**0.30**
**PBAT/PLA BC20**	**7.12**	**2.09**
**PBAT/PLA BC30**	**6.36**	**0.78**
**PLA/P(3HB-*co*-4HB)**	**4.47**	**1.19**
**PLA/P(3HB-*co*-4HB) BC10**	**2.84**	**0.14**
**PLA/P(3HB-*co*-4HB) BC15**	**2.48**	**0.45**
**PLA/P(3HB-*co*-4HB) BC20**	**2.25**	**0.28**
**PLA/P(3HB-*co*-4HB) BC30**	**1.61**	**0.22**
